# Conceptualisation of event roles in L1 and L2 by Japanese learners of English: the effect of perspectives of event construal on recognition memory

**DOI:** 10.1007/s00426-025-02190-5

**Published:** 2025-10-18

**Authors:** Jiashen Qu, Koji Miwa

**Affiliations:** Nagoya University, Nagoya, Japan

**Keywords:** Event roles, Recognition memory, Subjective construal, Thinking for speaking, Japanese, English

## Abstract

The previous studies on the interface of language and thought showed that event role hierarchies are similar across different languages, despite the different linguistic encodings (Ünal et al. (*Developmental Science*, *24*(6), e13116, 2021b); Isasi-Isasmendi et al. (*Open Mind,* *7*, 240–282, 2023)). However, Qu and Miwa (*Cognitive Linguistics, 35*(4), 547–577, 2024) observed that Japanese speakers prioritise animacy over agency, whereas English speakers prioritise agency in the linguistic encodings of event roles, reflecting the different preferences of the two languages for the degree of egocentricity in event construal. This study conducted an image memorisation experiment to investigate how these linguistic differences affect recognition memory of event roles. We found that Japanese speakers were more accurate in remembering human entities and showed no disadvantage in memorising non-human agents compared to English speakers, demonstrating an additive effect of animacy and agency. Additionally, Japanese speakers’ English proficiency influenced the degree of cognitive restructuring of event roles. This is the first study to present positive evidence for linguistic effects on the recognition memory of event roles, challenging the universality of conceptual knowledge of event roles across different languages.

## Introduction

Do speakers of different languages perceive the world in different ways? This question, termed *linguistic relativity* has been hotly debated for nearly a century. The recent decades have witnessed the birth of a series of competing proposals on the relationship between language and thought, discussing how deep and long-lasting the linguistic influence is on thought (for specific discussions, see Everett, 2013; Lucy, 2016; Wolff & Holmes, 2010). Although the debate has not reached a consensus, there is growing support that language influences thought by facilitating certain types of thinking (e.g., Divjak et al., 2020; Gleitman & Papafragou, 2005; Nedergaard et al., 2023; Ünal & Papafragou, 2016). One hypothesis that has drawn the core attention of researchers is the Thinking for Speaking (TFS) hypothesis (Slobin, 1996, 2004), which posits that we fit our thoughts into the encodable linguistic means when using language.

One of the most frequently tested domains for the TFS hypothesis is events. Events are coherent segments of activities with a discernible start and end in a specific spatial context. They are parsed from the continuous flow of daily activities and experiences, which is a fundamental aspect of human cognition (Zacks & Tversky, 2001; Kurby & Zacks, 2008). If speakers of different languages are equipped with different linguistic devices to describe the same events, do their mental representations of the same events vary? This question has been widely tested for various types of events in both L1 speakers and L2 learners. The research outcomes suggested that the available linguistic devices in one language augment the salience of the corresponding concepts, thus guiding speakers to pay more attention to and memorise the concepts more accurately when speakers are engaged in using languages either implicitly or explicitly (e.g., Fausey et al., 2010; Filipović, 2011; Flecken et al., 2014, 2015; Koster & Cadierno, 2019; Papafragou et al., 2008). Moreover, the language-specific TFS in L1 is entrenched and hard to be restructured for L2 learners (e.g., Cadierno, 2017; Filipović, 2016; von Stutterheim & Nüse, 2003), but restructuring is still possible, depending on L2 proficiency, age of acquisition, the context of acquisition, and length and frequency of linguistic exposure, etc. (Jarvis & Pavlenko, 2008; Pavlenko, 2014).

The present study extends the previous line of research and further investigates whether the TFS hypothesis holds for the domain of event roles. Event roles are the core components of events, which refer to the thematic roles assigned to noun phrases regarding a governing verb (Fillmore, 1968; Jackendoff, 1972, 1990). They carry the information of “who acted upon whom.” For example, in the sentence *I kicked the ball*, “I” is the agent, which initiated the action “kick,” and “ball” is the patient, which received the action. Several studies have explored whether different linguistic encodings of event roles affect the mental representations of event roles (Flecken et al., 2019; Isasi-Isasmendi et al., 2023; Ünal et al., 2021b). They all argued that the mental representations of event roles are similar across different languages. However, we suspect that the previous finding may not be generalisable to the Japanese language because Japanese speakers describe event roles with a subjective construal (Qu & Miwa, 2024), which is pervasive in the whole language and rarely observed in other languages (Ikegami, 2005). This cross-linguistic difference may have a greater impact on cognitive representations of event roles compared to the cross-linguistic differences examined in the previous studies. We will specify this point in the section below as we move on to the introduction of the cross-linguistic differences in event roles between Japanese and English. Furthermore, if the mental representations of event roles of Japanese speakers are different from the speakers studied before, it is worth probing whether and if so, how L2 learning experiences change the mental representations of event roles of Japanese speakers. To our knowledge, no study has explored this issue so far. In the section below, we first review the literature on the linguistic expressions and the mental representations of event roles before we introduce the details of the present study.

### Linguistic theories of event roles and the perspectives of event construal

An influential linguistic theory known as the Thematic Hierarchy (Grimshaw, 1990; Jackendoff, 1990; Pinker, 1989) ranks agent before patient based on the salience of the thematic roles. The more salient a thematic role is, the higher the syntactic position it is assigned to. Specifically speaking, compared to patients, agents are more frequently encoded as subjects. This agent-first assumption has been widely documented in the literature of psycholinguistics (e.g., Bock, 1986; Dowty, 1991; Gleitman et al., 2007; Prat-Sala & Branigan, 2000; Sauppe et al., 2023). Apart from agency, another important factor that influences the selection of subjects is animacy. Animate entities are more likely to be assigned with sentence subjects, compared to inanimate entities (e.g., Branigan et al., 2008; Ferreira, 1994; Gennari et al., 2012; Malchukov, 2008; Tanaka et al., 2011).

In a sentence, the syntactic subject is the focal point of the conveyed information (Bernolet et al., 2009; Lambrecht, 1994), so the syntactic subject reflects the perspective that the speaker takes on the event (Rissman et al., 2019). Although both agency and animacy influence whether the speaker assigns the syntactic subject to an agent or a patient, the extent of their impact varies across different languages, because speakers of different languages take different perspectives on the same event.

Japanese is argued to be a language that favours a subjective perspective in construing events, whereas Western languages, such as English, favour an objective perspective (Ikegami, 2005, 2008; Nakamura, 2004, 2009; Uehara, 2006). The notion of subjective construal and objective construal was first proposed by Langacker (1985, 1990), who highlighted the egocentricity in viewing events. He illustrated this notion through the examples below (Langacker,^1990^, p.20).

(1) *Vanessa is sitting across the table from me.*

(2) *Vanessa is sitting across the table*.

The distinction between the two sentences lies in the linguistic encoding of “me.” When “me” is explicitly expressed, (1) implies that the speaker maintains a detached viewpoint and perceives his involvement in the scene from an external standpoint. By splitting himself and positioning himself as an active participant, the speaker interprets the event from an outside vantage point, a more objective stance. Conversely, the absence of “me” in (2) suggests that the speaker embeds him/herself in the scene and views his/her participation in the scene from an immersed vantage point, a more subjective stance. Thus, the speaker is less conscious of his/her existence in (2) compared to (1). The unprofiled self in (2) indicates that the speaker interprets the event from an egocentric, subjective perspective.

The pronoun omission like (2) is prevalent and pragmatically natural in the Japanese language. Japanese speakers often avoid using first-person pronouns in natural conversations (Kuno, 1978; Lebra, 1992; Shibatani, 1990) and the pronoun omission extends to other persons (Ikegami, 2008), although Japanese lacks verb inflections that indicate pronoun reference. This indicates that a subjective perspective is strongly favoured in the Japanese language. In contrast, English speakers rarely omit pronouns (Uehara, 1998), which reflects a preference for objective construal. Moreover, reflexive expressions are different in Japanese and English. The translation equivalences of many reflexive constructions in English are either transitive verbs + body part or intransitive constructions in Japanese (Hirose, 1997; Ikegami, 2005), such as *hang oneself* – *hang neck*, *shave oneself* – *shave beard*, *behave oneself* – *behave*, *worry oneself* – *worry.* This sharp contrast further demonstrates the different preferences for the perspective of event construal in the two languages.

The cross-linguistic differences in the preferred perspectives between Japanese and English, as discussed above, pertain primarily to situations in which the speaker is directly involved in the event. A more outstanding difference is embodied in situations in which the speaker is not directly involved, which is of higher relevance to the focus of the present study. The preference for the subjective construal in Japanese encourages its speakers to mentally project themselves onto the scenes that they are to construe when they are not directly involved in the scenes (Ikegami, 2008). Consequently, Japanese speakers are prone to blend their viewpoints with those of the event participants and narrate the events from the perspective of the event participants, which is an internal perspective. When Japanese speakers do so, they are emotionally involved in the events, in which the level of empathy plays an important role (Kuno, 1978, 1987). This is because it is easier to project oneself onto an entity that one can easily empathise with than onto an entity that one cannot. The level of empathy follows the hierarchy below (Langacker, 1991, p.307).

(3) human > animal > physical object > abstract entity.

Importantly, this hierarchy, to a large extent, overlaps with the animacy hierarchy proposed by Siewierska (2004) and Silverstein (1986), probably because we evaluate the level of empathy by considering the similarities between us humans and other entities.

Therefore, when Japanese speakers are to select entities as subjects of sentences, they prioritise animacy over agency by taking an internal standpoint (i.e., subjective construal) with emotional involvement. For example, when Japanese speakers narrate a storybook, they tend to maintain a fixed viewpoint (usually that of the protagonist, the most animate entity in the story) throughout the narrative, irrespectively of whether that entity is acting as an agent or not in a given event (Nakahama, 2009; Nakahama & Kurihara, 2006). Another representative example comes from Ito (2016, 2018), who demonstrated that when describing images showing animal agents acting upon human patients (e.g., *A shark is chasing a man*), Japanese speakers tended to choose human patients as the subjects of sentences (e.g., *A man is escaping away from a chasing shark*), because the human patients are more animate than the animal agents. Moreover, the low acceptability of assigning inanimate agents the grammatical function of subjects in the Japanese language also reflects Japanese speakers’ preference for animacy over agency (VanArsdall & Blunt, 2022; Wolff & Ventura, 2009; Uchiyama, 1991). Similarly, Japanese learners of English have also been reported to have a low acceptability of and acquisition difficulty in English inanimate subjects (Master, 1991; Otaki & Shirahata, 2017; Shirahata et al., 2019).

Compared to Japanese, a more balanced emphasis is placed on agency and animacy in selecting events roles as sentence subjects in English. By taking an external standpoint (i.e., objective construal), English speakers take a bird’s eye view of the events (Ikegami, 2008), focusing more on objective facts of the events with less emotional involvement. Since the agent, as the initiator of the force chain, is the most outstanding and straightforward fact to capture from the event, the agent is often preferred by objective construal. For example, English speakers often change their viewpoints in the narratives based on the agent in a given event (Nakahama, 2009; Nakahama & Kurihara, 2006). In Ito (2016, 2018), English speakers tended to choose agent animals as the subjects of sentences. Moreover, English shows a high level of tolerance for inanimate agents serving as grammatical subjects, as seen in a sentence like *The wind blew the boy away* (Wolff & Ventura, 2009; Uchiyama, 1991). All these findings suggest that agency is highlighted in the English language (Bohnemeyer et al., 2010; Choi, 2009; Yamamoto, 1999, 2006). At the same time, animacy also plays an important role for English speakers. Although English speakers are more tolerant of inanimate agents, they do not actively select inanimate agents for subjects in language production (Bock, 1986; Hundt, 2004; Yamamoto, 1999). The agent bias in the English subject selection is predominantly seen in events with animate agents, whereas in cases that involve inanimate agents (e.g., natural disasters), this bias is considerably absent (Qu & Miwa, 2024).

The cross-linguistic differences between Japanese and English in linguistic encodings of event roles were systematically tested in an experimental study by Qu and Miwa (2024). We asked participants to describe line-drawn images showing agents acting upon patients (e.g., a tiger is chasing after a boy). The target images consisted of four categories: humans throwing objects, humans chasing animals, animals chasing humans, disaster threatening humans. A significant cross-linguistic difference was found in the category of animals chasing humans: Japanese participants chose human entities as the subjects of sentences most of the time whereas English participants often chose animals as the subjects. The authors argued that this cross-linguistic difference demonstrated that animacy and agency affect the linguistic encodings of event roles in different ways in the two languages. This can be attributed to the different preferences for the degree of egocentricity in the perspective of event construal in Japanese and English.

### The mental representations of event roles across different languages

The research on the interface of language and thought in event representations argues for a tight link between how people describe events and how they cognitively represent events. Several articles reviewed an expanding collection of empirical findings that explored the interface between language and thought in event construal (e.g., Papafragou & Grigoroglou, 2019; Ünal et al., 2021a), and they claimed that a similar structure is shared between the mental representations of events and the linguistic expressions of events, though the structure is not strictly the same between the two. The previous studies suggested that event roles belong to part of this picture.

The studies on event role representations generally suggest that although human beings can quickly extract the information of both agents and patients (Hafri et al., 2013, 2018; Rissman & Majid, 2019), agents are more salient than patients. Agents are often attended to more rapidly and thoroughly than patients during the event perception (Cohn & Paczynski, 2013; Gerwien & Flecken, 2016; Isasi-Isasmendi et al., 2023; Wilson et al., 2011, 2014), though this is not always the case depending on the task demands (Ünal et al., 2021b). For example, Ünal et al. (2021b, 2024) found that the colour changes in agents were detected more slowly and less accurately than those in patients in a change blindness task. They attributed the result to the fact that the colour change is the property change for the agent rather than the agent change itself. Therefore, the findings from the prior studies, in general, are consistent with the thematic hierarchy, which places the agent at the top, followed by the patient.

More importantly, these findings were not restricted to the English language. The studies which focused on other languages, such as Basque and Spanish (Isasi-Isasmendi et al., 2023), Chinese (Flecken et al., 2019) and Turkish (Ünal et al., 2021b), all claimed that despite the cross-linguistic variation in expressing event roles (e.g., the agents drop in Chinese and Turkish), the mental representations of event roles are similar among speakers of different languages. This further indicates that the conceptual knowledge of event categories is universal across speakers of different languages (Gleitman & Papafragou, 2005; Ünal & Papafragou, 2016), which is not in line with the alternative view that cross-linguistic differences give rise to cognitive biases when viewing the world (Boroditsky, 2001, 2006; Levison, 2003).

However, the conclusion of a cognitive universal for event roles is premature for two reasons. First, theoretically speaking, because the TFS hypothesis predicts a close connection between conceptual representations and linguistic encoding of concepts, it is reasonable to hypothesise that cross-linguistic differences in linguistic expressions of event roles can modulate cross-linguistic differences in the salience of the mental representations of event roles at least when language is involved in solving cognitive tasks. Although the previous findings seem to have disproved this theoretical possibility, this theoretical possibility is still worth testing because only a small number of linguistic features were investigated. It is of particular interest to focus on the linguistic devices that are potentially more influential on thought than the previously studied ones because different aspects of language may exert qualitatively different effects on thought, such as lexical vs. grammatical (Lucy, 2016). This leads us onto the second point. Second, typologically speaking, the Japanese language is distinct from many languages in terms of the linguistic encodings of event roles. The subjective construal favoured by Japanese is pervasive and reflected at many linguistic levels (See Ikegami, 2005, for detailed discussions). As introduced in 1.1, the preference for a subjective perspective encourages Japanese speakers to project themselves onto the most animate entities in event construal, which gives rise to an animacy advantage over agency when selecting entities as subjects of sentences in Japanese. This advantage can arguably guide Japanese speakers to memorise human patient entities more accurately than non-human agent entities, which can be tested using images categories such as animals chasing humans, as in Qu and Miwa (2024). This theoretical possibility directly questions the hypothesis of the agent preference as a cognitive universal (e.g., Isasi-Isasmendi et al., 2023; Jackendoff, 1990; Rissman & Majid, 2019), therefore testing the validity of the claim that conceptual knowledge of event roles is universal across languages as well as calling for research on more diverse languages in the cognitive science (Blasi et al., 2022; Henrich et al., 2010; Majid & Levinson, 2010).

### L2 learning and cognitive restructuring

Because there is a close connection between linguistic expressions and concepts, learning a new language indicates not only the acquisition of linguistic forms but also the modification of underlying concepts. This gives rise to an interesting question: Does learning a second language change one’s cognitive patterns?

A large body of L2 studies examined this research question by focusing on different semantic domains (e.g., see Athanasopoulos et al., 2011 for colour perception; Pavlenko & Malt, 2011 for object perception; Filipović, 2011 and Ji, 2017, 2019 for motion perception; Alvarado & Jameson, 2011 for emotion processing). Although the findings are not fully conclusive, the studies reported that L2 learning can shift the cognitive patterns of L2 learners towards those of L1 speakers (Jarvis & Pavlenko, 2008; Pavlenko, 2011, 2014).

Several factors contribute to such cognitive restructuring. Bylund and Athanasopoulos (2014) provided an overview of these crucial factors that underlie the degree of cognitive restructuring in L2 learners. They are language proficiency (Athanasopoulos, 2006, 2007; Ji, 2017), language contact (Bylund et al., 2013; Wang & Wei, 2019), context of acquisition (Kurinski & Sera, 2011; Montero-Melis et al., 2016; Koster & Cadierno, 2019), age of acquisition (Athanasopoulos & Kasai, 2008; Boroditsky, 2001; Kersten et al., 2010), bilingual language mode (Kersten et al., 2010), and the length of immersion in L2 (Athanasopoulos, 2009; Park, 2020).

However, some studies reported no cognitive restructuring in L2 learners, suggesting that cognitive restructuring is conditional or challenging for L2 learners (e.g., Flecken et al., 2015; Filipović, 2011, 2016; von Stutterheim & Carroll, 2006; Schmiedtová & Sahonenko, 2012; Schmiedtová et al., 2011). The possible reason is that L2 learners may rely on their L1 conceptual categories when solving cognitive tasks. Due to the differences in the acquisition processes of L1 and L2, the linguistic structures of later-learned languages are often disconnected from their cognitive and social functions in bilingual speakers (Jarvis & Pavlenko, 2008).

To the best of our knowledge, no study has explicitly investigated how L2 learners acquire the conceptual categories of event roles different from their L1, such as those between Japanese and English as introduced in 1.2. More importantly, the present study that focuses on how Japanese learners of English restructure the mental representations of event roles in L2 is distinct from many previous studies, in that (1) the conceptual categories of agents and patients go beyond one-to-one mapping between a linguistic label and a percept (e.g., colour terms, temporal terms) to more complicated relations between the two entities (Flecken & Van Bergen, 2020), (2) the cross-linguistic differences in conceptualising event roles between Japanese and English are not all-or-none but rather probabilistic. The probabilistic cross-linguistic difference in expressing event roles may pose great learning difficulty for Japanese learners of English due to low noticeability (Qu & Miwa, 2024). Japanese learners need to receive enough amount of English input to raise their awareness of how event roles are conceptualised differently in English. The acquisition difficulty in the linguistic expressions of event roles indicates that Japanese learners might have great difficulty in reconceptualising their cognitive representations of event roles in L2 English.

### The present study

To address the open issues mentioned above, the present study investigated whether the cross-linguistic differences in the expressions of event roles driven by the perspective of event construal (subjective vs. objective) guide speakers’ mental representations of event roles. We approached the mental representations of event roles by testing recognition memory of event roles. Recognition memory is a kind of declarative memory, which requires the ability to both identify and judge whether a given stimulus matches a previously encountered stimulus (Mandler, 1980). Accuracy and speed in recognition memory tasks can be improved through the learning of categories (Shiffrin & Schneider, 1977). Language, as a powerful tool for categorisation, can exert a significant influence on recognition memory (Lupyan et al., 2020). Therefore, in the present study, we chose recognition memory as a window into exploring the relationship between linguistic expressions of event roles and their mental representations. We focused on accuracy data over reaction time data in the present study, the decision of which was based on both theoretical and methodological considerations. First, although Boroditsky (2001) argued that implicit measures (e.g., reaction time) are more convincing as the evidence for language-on-cognition effects than explicit measures (e.g., making judgments), our theoretical interest lay in whether there are cross-linguistic differences between Japanese and English participants in their outcomes of recognition memory of event roles. By observing whether one group of speakers remembers a particular part of an event more accurately than another group, we can present more explicit (counter)evidence for the linguistic relativity hypothesis: speakers of different languages see the world differently. In this sense, response accuracy is a more straightforward measure than response time. Second, methodologically speaking, response time can be more sensitive and susceptible to experimental settings than accuracy, especially in online experiments, in which devices can vary from participant to participant. Thus, accuracy analysis in the present online experiment was arguably a more suitable approach for investigating the cross-linguistic differences in recognition memory of event roles.

**Fig. 1:**
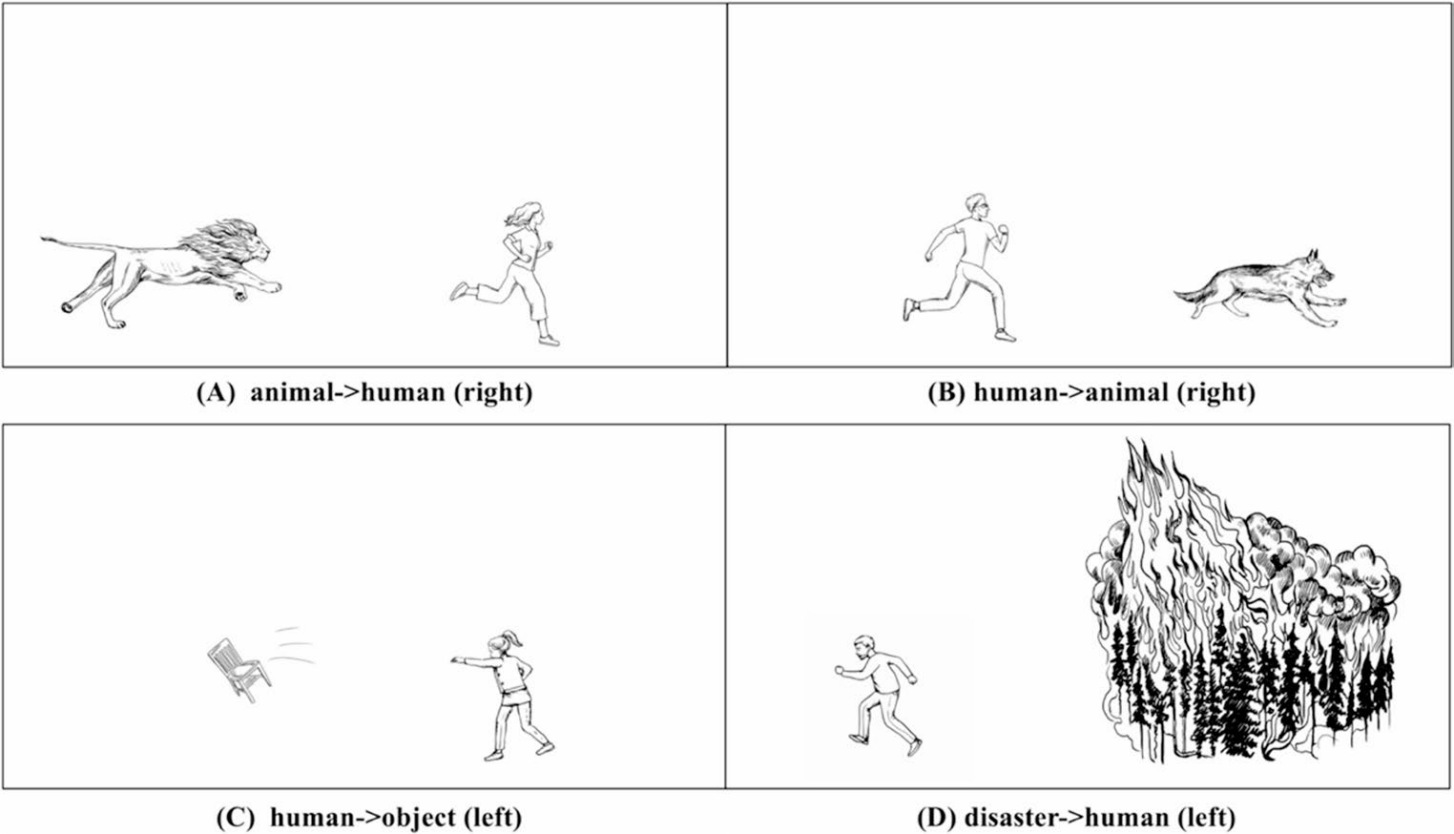
The four categories of images in Qu and Miwa (2024). The direction in the parentheses indicates the direction of actions

We had two research questions in the current study. First, we studied whether the recognition memory of event roles differs between Japanese and English speakers. Based on a previous study on the cross-linguistic differences in the linguistic expressions of event roles between the two languages (Qu & Miwa, 2024), we hypothesised that, influenced by animacy, Japanese speakers are more accurate in memorising human entities than English speakers when human entities are patients, such as the human entities in “animals chasing humans” (panel A in Fig. 1) and “disasters threatening humans” (panel D in Fig. 1), particularly in “animal chasing humans”. When human entities are agents (panels B and C in Fig. 1), we predicted no cross-linguistic differences between Japanese and English speakers because both languages prioritise human agents.

In contrast, English speakers, influenced by agency, are more accurate in memorising non-human agents than Japanese speakers, such as the animal entities in “animals chasing humans”. But this is further mediated by the animacy of non-human agents: the advantage in memorising non-human agents is more likely to appear when non-human agents are animate (e.g., animal agents > disaster agents), for animacy is also an important factor for English speakers in memorising event roles.

The second question is whether and if so, how L2 learning experiences change the patterns of recognition memory of event roles by Japanese learners of English. We hypothesise that L2 learning experiences can partially weaken the animacy effect and enhance the agency effect in Japanese learners of English when recognising event roles, thereby aligning their performance more closely with that of native speakers of English.

### Predictors considered in this study

We were interested in the following predictors that might influence the recognition memory of event roles for Japanese and English speakers. Table 1 summarises the predictors considered in the present study.

The first predictor *language* has two levels: L1 English and L1 Japanese. The second predictor *category* refers to the four categories of the images. The third predictor *pairType* refers to the four types of relationships between Image 1 and Image 2 with respect to what is not matched/unmatched between Image 1 and Image 2. Apart from these predictors, we also included several task-related predictors (*trials*,* direCongru*,* image1Dur*,* waitingDur*) as well as the participant-related predictors (*LexTALE_score*, *averageEngs*, *foreign_experience*, *exposureEng*,* digit_score*). The predictor *trials* indicates the *n*th trial count throughout the experiment. The predictor *direCongru* indicates whether the direction of the agent-patient action chain shown in Image 1 is consistent with that in Image 2 (e.g., *congruent* when the agent acted upon the patient from left to right for both Image 1 and Image 2). The predictor *image1Dur* indicates the presentation time of Image1, ranging from 700 ms to 3000 ms, and the predictor *waitingDur* indicates the length of waiting time, ranging from 1000 ms to 8000 ms. We were interested in how the presentation time of stimuli (Image 1) as well as the interstimulus interval between Image 1 and Image 2 (target) influence the recognition memory of the images. The predictors *LexTALE_score* and *averageEngS* indicate the English proficiency of participants. LexTALE is an unspeeded lexical decision task (Lemhöfer & Broersma, 2012). *The LexTALE_score* was automatically calculated based on the number of correct responses in judging whether a word is an existing English word, the range of which is 0–100. *averageEngS* was calculated by averaging the three self-reported scores of L2 proficiency in LEAP-Q: L2 listening, L2 reading, and L2 speaking. The predictor *digit_score* indicates the verbal working memory scores of participants measured by the digit span test, which might influence the speed and accuracy of retrieving the memory of Image 1.

**Table 1:** Predictors considered in this study. The means were calculated before the standardisation procedure

Predictors	Range	Levels, Mean (SD)
language		L1English, L1Japanese
category		animal->human, human->animal, human->object, disaster->human
pairType		Identical, AgentUnmatch, PatientUnmatch, Nonmatch
trials	1: 200	100.5 (57.88)
direCongru		congruent, incongruent
Image1Dur	0.7: 3	1.85 (0.69)
waitingDur	1: 8	4.49 (2.29)
LexTALE_score	43.75: 77.50	60.70 (7.20)
averageEngS	1: 9.67	5.43 (2.13)
digit_score	3: 8	6.05 (1.63)

## Experiment: image memorisation experiments

### Method

#### Participants

Forty-eight native speakers of Japanese participated in the experiment (females = 26, mean age = 24, *SD* = 4.81). They were recruited from several Japanese universities through a Japanese participant recruitment website (https://www.jikken-baito.com/). All the Japanese participants were Japanese-English late bilinguals, who started to learn L2 English sometime after five years old at school. Sixty-one native speakers of English were recruited on Amazon Machinal Turk (female = 22, mean age = 33, *SD* = 4.99). They all reported that they either spoke only English or had very limited knowledge of other languages, such as Spanish, Mandarin, including Japanese. All participants filled a consent form on Google Forms before the experiments. Our experiment involved new task manipulations and tested new languages that had not been tested in past studies. Our analyses included interactions between two factors (language and category) with two and four levels respectively. For these reasons, although we ensured a greater number of items and participants than those tested in past studies, it was challenging to estimate the required sample size precisely in advance. Therefore, we conducted post-hoc power analyses with the *SIMR* package (Green & MacLeod, 2016), following the approach taken in some past studies (e.g., Antal & de Almeida, 2024), on a subset of our full dataset (*PatientUnmatch* in animal->human). We ran 1000 simulations to estimate the minimum number of participants needed to achieve 80% power within a 95% confidence interval. The simulation results indicated that a total of 80 participants (40 participants in each language group) would reach the power threshold. Because the current study recruited a total of 109 participants, it had sufficient statistical power.

#### Materials

We opted for the line-drawn materials used in Qu and Miwa (2024). The materials consisted of 200 images that all depicted an action caused by an agent upon a patient. There were four categories of images, with 50 images in each of the following four categories: “animals chasing humans,” “humans chasing animals,” “humans throwing objects,” and “disasters threatening humans.” Each image had two versions with respect to the direction of agent-to-patient actions: right and left. See Fig. 1 for sample images.

#### Procedure

The experiment was conducted online. Participants were directed to an experiment webpage created by the authors and participated in the experiment by following the instructions written on the webpage. They were asked to participate using a computer (rather than a smartphone or a tablet) in a quiet environment with a stable Internet access. The instructions throughout the whole experiment were either in English for English participants or in Japanese for Japanese participants.

Participants first took part in an image memorisation experiment. The image memorisation experiment was programmed in PsychoPy (Version 2022.1.1, Peirce et al., 2019) and hosted on an online platform Pavlovia (http://pavlovia.org/). In the image memorisation experiment, two images were presented sequentially. Participants were asked to judge whether the second image with a red border is the same with the first image they saw before. If the two images were the same, they were expected to press the “J” key, otherwise the “F” key. Participants were instructed to look at the fixation mark before the first image appears. The fixation mark lasted for 1000 ms, and it was placed at the left side of the screen instead of the middle of the screen to make sure that participants perceived the left image first. The duration of the first image presentation was set randomly and ranged from 700 ms to 3000 ms at 100 ms intervals. After participants saw the first image, they had to wait until the second image appeared. The waiting time (i.e., the duration between the offset of the first image and the onset of the second image) was set randomly and ranged from 1000 ms to 8000 ms at 1000 ms intervals.

We randomised the durations of Image 1 and the waiting screen for two reasons. First, since static images are used as experimental stimuli, randomised durations can arguably prevent temporal predictability and therefore encourage participants to engage with dynamic event representations. A better simulation of event dynamicity can increase cognitive demand and thus further encourage participants to adopt language-induced strategies rather than superficial visual strategies in memorising events, which is beneficial for studying the mapping between language and cognition. Second, by manipulating the duration of stimulus exposure and inter-image intervals, the design enables us to examine how the magnitude of inner language engagement influences the robustness of event encoding (Image 1) and information storage before recall (waiting time) respectively. This is consistent with predictions from the linguistic bootstrapping hypothesis (LBH), which maintains that inner language is beneficial for both encoding the event information and recalling the memorised event information (Banks & Connell, 2024; Connell, 2019; Connell & Lynott, 2014; Dymarska et al., 2022). The role of waiting time is particularly interesting. The randomised duration of waiting time as a continuous variable might have different interactions with Japanese and English during the information storage because the inner language strategies adopted to hold the encoded information of event roles in memory are arguably different between the two languages. Therefore, we may observe two distinct temporal courses in how the memory accuracy for event roles changes in Japanese and English during waiting time. This novelty over the previous studies can deepen our understanding of “Thinking for Speaking.”

**Fig. 2:**
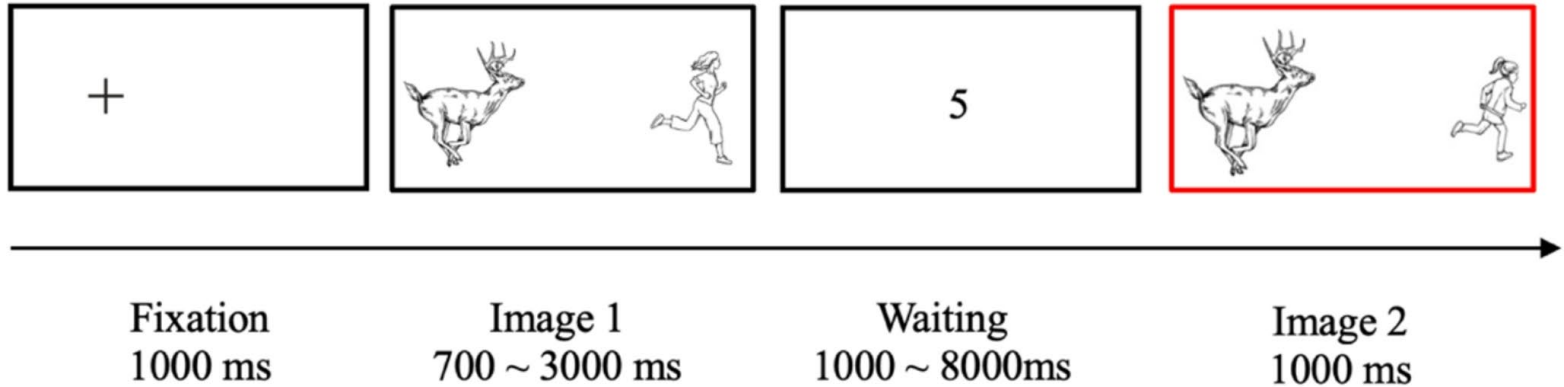
A sample trial (*PatientUnmatch*) in the image memorisation experiment

A timeout screen appeared when participants did not respond within 8000 ms. See Fig. 2 to see a sample trial in the image memorisation experiment.

There were four types of relationships between Image 1 and Image 2: (1) *Identical*, (2) *AgentUnmatch*, (3) *PatientUnmatch*, and (4) *Nonmatch*. *Identical* means that the two images were the same regardless of the direction. We asked participants not to take the direction into consideration when judging whether the two images were the same. This was to motivate participants to memorise the relationship between the two entities beyond visuo-perceptual memorisation of the whole image. *AgentUnmatch* means that the agent in Image 2 was different from that in Image 1. *PatientUnmatch* means the patient in Image2 was different from that in Image1. *Nonmatch*^1^ means that neither the agent nor the patient was the same as those in Image 1. However, Image 1 and Image 2 were always matched in category. That is, if Image 1 shows an image of animal->human, Image 2 will also present an image of animal->human.

In total, there were 200 trials in the image memorisation experiment, out of which 100 trials were for *Identical*, 40 trials for *AgentUnmatch*, 40 trials for *PatientUnmatch*, and 20 trials for *Nonmatch*. The total number of trials for “yes” was equal to that for “no.” After every 30 trials, participants were given feedback with their average reaction time and accuracy.

After the image memorisation experiment, participants completed a digit-span task. The digit span task was programmed in PsychoPy and hosted on Pavlovia. Digit span tasks are the most widely used psychological tests to measure verbal working memory (Richardson, 2007). We programmed a backward digit span task, where participants were shown a sequence of numbers on the screen, one number at a time. Then, they were asked to type down the sequence of numbers in the reversed order. The length of the sequence of numbers started from three digits and gradually increased by one digit after every three trials until it reached eight digits. Participants could move onto a longer digit only if they got at least two out of three trials of the current digit correct.

After the digit span task, participants performed LexTALE, which is a 5-minute unspeeded lexical decision task to measure the vocabulary knowledge of L2 learners (Lemhöfer & Broersma, 2012). We programmed the test in PsychoPy and hosted it on Pavlovia.

Finally, participants answered a language experience and proficiency questionnaire called the LEAP-Q (Marian et al., 2007), which was adopted in Google Forms by the authors. Japanese participants answered the questionnaire in Japanese, and English participants in English.

## Results and discussion

The data analyses were mainly focused on accuracy because participants’ likelihood of making categorical decisions (i.e., *yes* or *no*) should help us to understand whether speakers of different languages conceptualize event roles differently (for RT analyses, see Appendix IV). Participants whose accuracies were below 57.5%, a cut-off line determined by an exact binomial test (Conover, 1971), were removed. Eventually, we had data for 49 English participants and 48 Japanese participants. We also removed the data points with RTs shorter than 300 ms or longer than 8000 ms (a timeout screen appeared after 8000 ms). The removed data points accounted for 0.38% of the whole data, leaving 19,519 data points for the accuracy analyses. All the data analysed in the present study can be found on the Open Science Framework: https://osf.io/q2eyf/files/osfstorage.

The average accuracy for each *pairType* across all participants was 87.5% for *AgentUnmatch*, 88.4% for *PatientUnmatch*, 90.4% for *Identical*, and 96.2% for *Nonmatch*, respectively (See Appendix VI for the detailed report). The fact that the average accuracy advantage for Nonmatch was the highest indicates that participants relied on both agents and patients to judge whether the current image matched with the previous one, which further suggests that participants processed both agents and patients during the task. Otherwise, there would be no difference between *Nonmatch* and *AgentUnmatch* or *PatientUnmatch*.

### Accuracy analyses for the cross-language TFS

We were most interested in whether there were differences in accuracy between Japanese and English participants in *AgentUnmatch* and *PatientUnmatch.*^2^ To simplify statistical models and achieve interpretive ease, we separately analysed the data for *AgentUnmatch* (3,906 data points) and *PatientUnmatch* (3,900 data points). We fitted generalised linear mixed-effects models (GLMM) with a logit link function to the data in R version 4.2.2 (R Core Team, 2022). The lme4 package was used for the GLMM (Baayen et al., 2008; Bates et al., 2015; Jaeger, 2008). The *lmerTest* package (Kuznetsova et al., 2017) was used to calculate p-values.

**Fig. 3:**
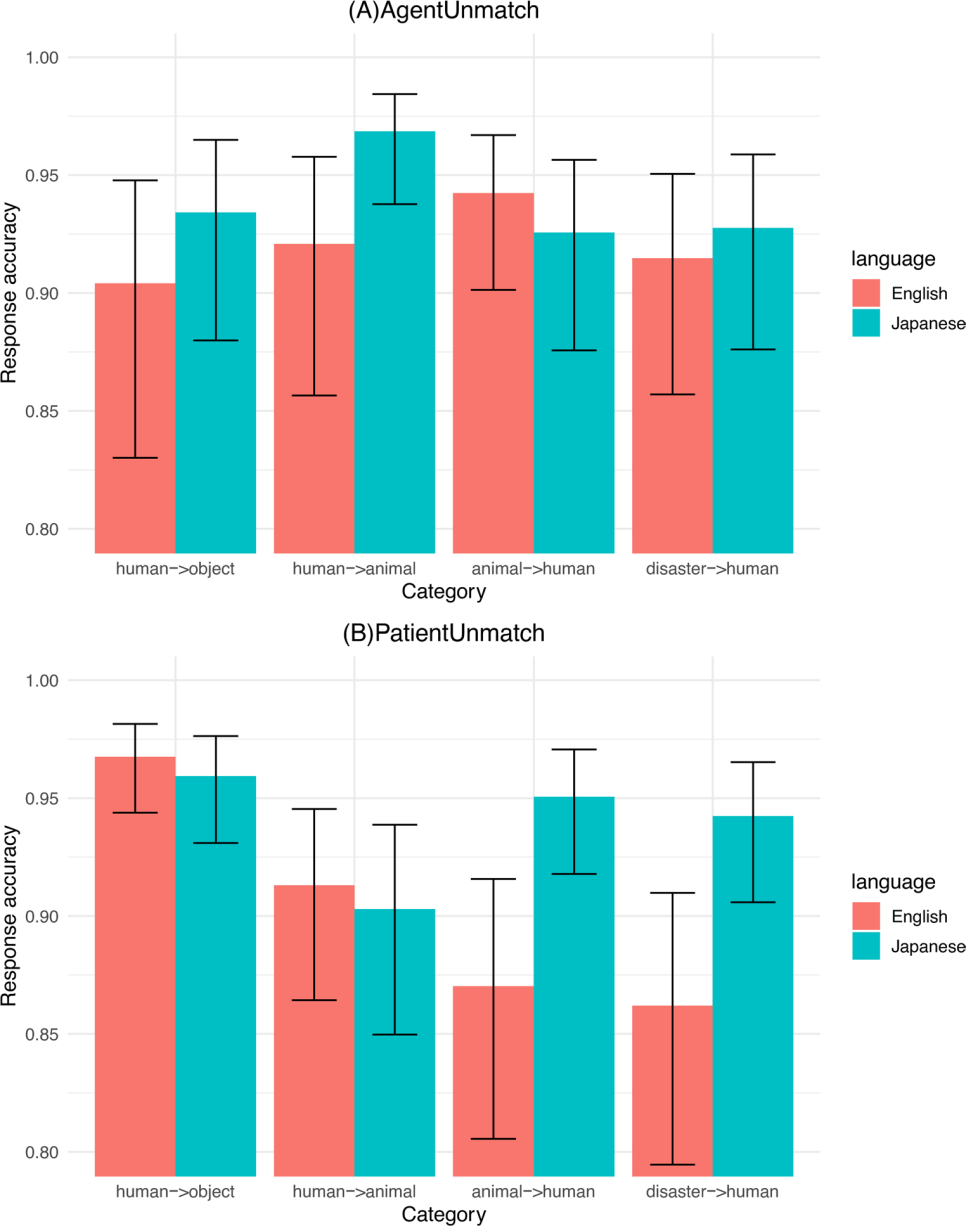
Response accuracies of *AgentUnmatch* (**A**) and *PatientUnmatch* (**B**) by the two language groups across the four categories of images

To set the proper random-effects structure, we first included *participant_ID* and *Image1* as the random effects. Then, we tested random slopes and random contrasts for all the predictors: *trials*, *category*, *direCongru*, *image1Dur*, *waitingDur*. We then simplified the random-effects structure by keeping only significant random-effect parameters. We included *language*, *category*, the interaction between the two, *direCongru*, *image1Dur*, *waitingDur*,* digit_score* as the fixed effects. All the categorical predictors were sum-coded. All the continuous variables were standardised.^3^ Table 2 summarises the list of fixed effects for the *AgentUnmatch* condition. Table 3 summarises the list of fixed effects for the *PatientUnmatch* condition. Figure 3 shows the response accuracies of *AgentUnmatch* (A) and *PatientUnmatch* (B) by the two language groups across the four categories of images.

From the two tables, we can first see that participants became more accurate as the trials increased, and the effect was more obviously seen in *AgentUnmatch* trials. Moreover, the response accuracy was significantly influenced by the presentation time of Image 1 in both *AgentUnmatch* and *PatientUnmatch* trials, which suggests that the longer the time given for information encoding, the more accurately the information was memorised. Interestingly, the effect of waiting time (the presentation delay between Image 1 and Image 2) was observed only in *AgentUnmatch* but not in *PatientUnmatch* trials, indicating that the memory decay for agents was more outstanding than that for patients. This result further implies that compared to the patients, participants may have first identified and memorised the agents. This is also indirectly evidenced by a negative effect of the direction congruency (*direCongru*) between Image 1 and Image 2 that was observed only in *AgentUnmatch*. Participants were expected to press the “no” button in both *AgentUnmatch* and *PatientUnmatch* trials, so the incongruent direction between the two images could arguably motivate participants to judge the target image to be different from the previously seen image, even though the direction did not matter in the experiment. Participants seemed to rely more on the incongruent direction between the images in *AgentUnmatch* than in *PatientUnmatch* trials, due to the faster memory decay for the agents.

**Table 2:** The fixed effects structure of the generalized linear mixed-effects model for the response accuracy of *AgentUnmatch* trials in the four categories^1^

	Estimate	Std.Error	z-value	*p*-value
(Intercept)	2.628	0.165	15.922	< 0.001
trial_s	0.118	0.055	2.151	0.031
language1	−0.161	0.128	−1.253	0.210
category1	−0.179	0.207	−0.868	0.386
category2	0.313	0.217	1.447	0.148
category3	0.031	0.224	0.138	0.890
Image1Dur_s	0.269	0.055	4.936	< 0.001
waitingDur_s	−0.171	0.056	−3.055	0.002
direCongru1	0.222	0.109	2.028	0.043
language1:category1	−0.044	0.108	−0.406	0.685
language1:category2	−0.326	0.118	−2.763	0.006
language1:category3	0.297	0.134	2.216	0.027

The R code was glmer(Correct/Incorrect ~ trial_s + language*category + image1Dur_s + waitingDur_s + direCongru + (1 + category | participant_ID) + (1 | Image1), data=dat_AgentUnmatch)

^1^language1 refers to English; category1 refers to human->object, category2 refers to human->animal, category3 refers to animal->human; direCongru1 refers to the incongruent direction between Image1 and Image2. these levels remained unchanged throughout the whole data analysis

**Table 3:** The fixed effects structure of the generalized linear mixed-effects model for the response accuracy of *PatientUnmatch* trials in the four categories

	Estimate	Std.Error	z-value	*p*-value
(Intercept)	2.573	0.147	17.510	< 0.001
trial_s	0.121	0.063	1.910	0.056
language1	−0.207	0.122	−1.696	0.090
category1	0.700	0.167	4.189	< 0.001
category2	−0.287	0.153	−1.869	0.062
category3	−0.148	0.156	−0.949	0.343
Image1Dur_s	0.199	0.055	3.600	< 0.001
language1:category1	0.325	0.111	2.933	0.003
language1:category2	0.268	0.090	2.979	0.003
language1:category3	−0.319	0.094	−3.385	< 0.001

The R code was glmer(Correct/Incorrect ~ trial_s + language*category + image1Dur_s + (1 | participant_ID)+(1 | Image1) + (0 + trial_s | participant_ID), data = dat_PatientUnmatch). trial_s was kept in the fixed-effects structure because the by-participant random slopes for trial_s were significant

Of our particular interest is the interaction of *language* and *category*. The significant interactions of *language* and *category* shown in the tables indicate that the two groups of participants memorised the agents and patients differently across the four categories. We further compared the magnitude of the cross-linguistic differences between the two language groups in the four categories, using the *emmeans* package (Lenth, 2023). In *AgentUnmatch* trials, a significant cross-linguistic difference was found in the category *human->animal* (*p* =.015), suggesting that Japanese participants were more accurate in detecting the change in the human agents. This is against our hypothesis that the two language groups would not differ in memorising the human agents. No significant differences between the two language groups were observed in the rest of the categories. The non-significant results for the categories of *animal->human* and *disaster->human* implied that Japanese participants memorised the non-human agents as accurately as the English counterparts, which is again inconsistent with our hypothesis that English speakers would remember the non-human agents more accurately than Japanese speakers.

In *PatientUnmatch* trials, the pairwise comparisons did not show significant cross-linguistic differences in the categories of *human->object* and *human->animal*, which suggests that the two language groups were similarly accurate in memorising the non-human patients. In contrast, Japanese participants were significantly more accurate in memorising the human patients than English participants in both the category of *animal->human* (*p* <.001) and the category of *disaster->human* (*p* =.001). Moreover, the magnitude of the cross-linguistic differences became larger as the waiting time became longer (See Appendix II), which serves as positive evidence for the TFS hypothesis. The results of *PatientUnmatch* are in line with our hypothesis that Japanese speakers would memorise human patients more accurately than English speakers.

### Accuracy analyses for the within-language TFS

**Fig. 4:**
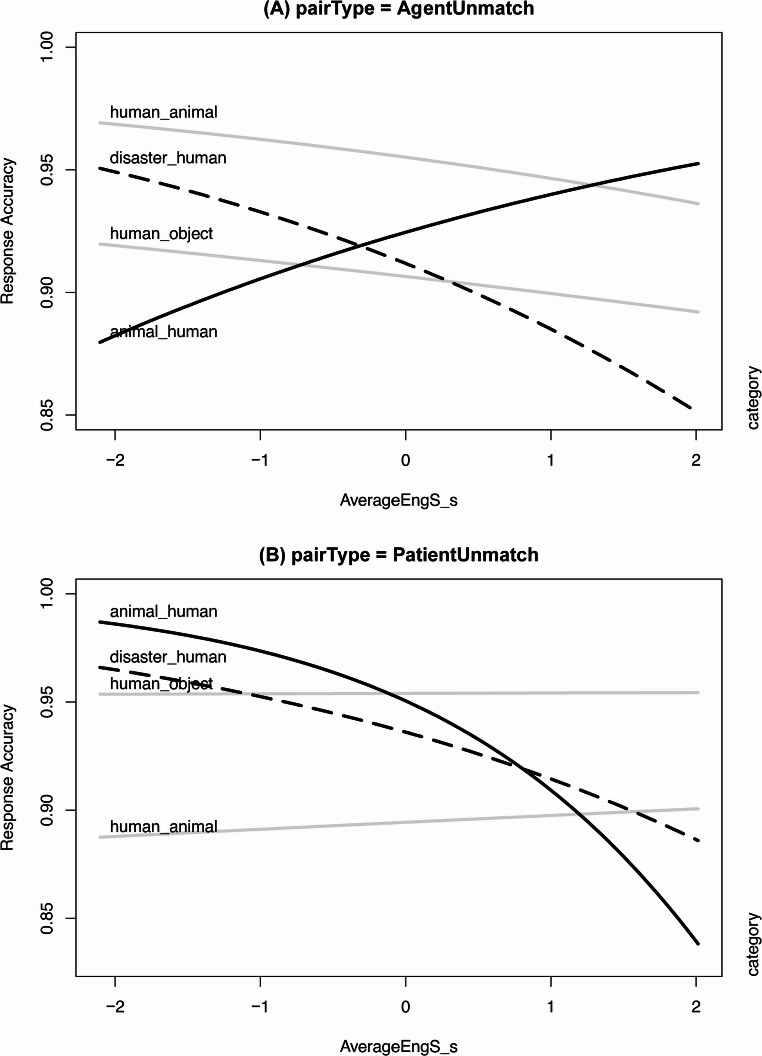
Japanese response accuracies in *AgentUnmatch* (**A**) and *PatientUnmatch* (**B**) trials across the four categories of images mediated by English proficiency

We further analysed the Japanese data to examine how L2-related predictors influenced the patterns of recognition memory of event roles. Following the data analysis above, we separately analysed the data for *AgentUnmatch* (1,919 data points) and *PatientUnmatch* (1,917 data points) trials. The optimal random-effects structure was decided after we tested random slopes and random contrasts for all the predictors. In addition to the fixed-effect variables considered in the analyses above, we added the following L2-related predictors one by one in the models to see which predictor significantly influenced the Japanese participants’ response accuracy: *LexTALE_score*, *averageEngS*. Table 4 summarises the list of fixed effects for Japanese participants’ response accuracy in *AgentUnmatch* trials. Table 5 summarises the list of fixed effects for Japanese participants’ *PatientUnmatch* trials. Figure 4 visualises the response accuracies of *AgentUnmatch* (Panel A) and *PatientUnmatch* (Panel B) by Japanese participants across the four categories of images.

**Table 4:** The fixed effects structure of the generalized linear mixed-effects model for the Japanese participants’ response accuracy of *AgentUnmatch* trials in the four categories

	Estimate	Std.Error	z-value	*p*-value
(Intercept)	2.560	0.177	14.472	< 0.001
AverageEngS_s	−0.079	0.150	−0.527	0.598
category1	−0.271	0.188	−1.443	0.149
category2	0.515	0.208	2.478	0.013
category3	−0.037	0.193	−0.191	0.848
Image1Dur_s	0.238	0.079	2.992	0.003
AverageEngS_s: category1	−0.001	0.132	−0.006	0.995
AverageEngS_s: category2	−0.107	0.163	−0.658	0.510
AverageEngS_s: category3	0.327	0.142	2.303	0.021

The final model for Japanese AgentUnmatch was glmer(Correct/Incorrect ~ averageEngS *category + image1Dur_s + (1 | participant_ID) + (1 | Image1), data = dat_JapAgentUnmatch)

**Table 5:** The fixed effects structure of the generalized linear mixed-effects model for the Japanese participants’ response accuracy of *PatientUnmatch* trials in the four categories

	Estimate	Std.Error	z-value	*p*-value
(Intercept)	2.701	0.178	15.146	< 0.001
AverageEngS_s	−0.234	0.164	−1.432	0.152
category1	0.330	0.178	1.858	0.063
category2	−0.565	0.155	−3.650	< 0.001
category3	0.253	0.181	1.399	0.162
AverageEngS_s: category1	0.238	0.166	1.439	0.150
AverageEngS_s: category2	0.268	0.135	1.990	0.047
AverageEngS_s: category3	−0.423	0.163	−2.596	0.009

The final model for Japanese participants’ response accuracy in PatientUnmatch trials was glmer(Correct/Incorrect ~ averageEngS *category + (1 | participant_ID) + (1 | Image1), data = dat_JapPatientUnmatch)

Here, we reported only the models for the self-reported average English scores (*averageEngS*) because it was the only predictor that showed significant interactions with *category* in both *AgentUnmatch* and *PatientUnmatch*. We also tested other L2 predictors, such as LexTALE scores, the daily exposure to English and the experience of living in English-speaking countries which were reported in Appendix III.

Based on the tables and the figure, we can see that in *AgentUnmatch* trials, compared to the other three categories, the category animal->human showed an upward trend as the L2 proficiency increased as indicated by a black solid line, which suggests that the L2 learning experience guided the Japanese speakers to be more sensitive to agency and thus better memorised the non-human animate agents. In contrast, in *PatientUnmatch* trials, the category animal->human showed a downward trend as the L2 proficiency increased as indicated by a black solid line, which implies that the L2 learning experience guided the Japanese speakers to be less sensitive to the human patients and thus showed an attenuated human-oriented perspective in memorising the human patients, which is also shown in the category of disaster->human as indicated by a black dashed line. The finding here is in line with our hypothesis that L2 learning experiences change the patterns of recognition memory of event roles by Japanese learners of English.

Surprisingly, in *AgentUnmatch* trials, the category of disaster->human showed a downward trend as the L2 proficiency increased as indicated by a black dashed line. One possibility is that Japanese participants with higher English proficiency might have been influenced by multiple language-induced strategies in encoding the event roles of this category. Specifically speaking, driven by the agency effect in L2 English, the Japanese learners of English with higher English proficiency should have paid more attention to the disaster agents than the learners with lower English proficiency. However, since the disaster entities are inanimate agents, the agency effect was smaller than that for the animate agents, such as the animals, which might have guided the Japanese learners with higher English proficiency to go back to the human patients. Switching back and forth between the disaster agents and the human patients may have diverted attention away from the disaster entities, according to the temporal decay hypothesis (Barrouillet et al., 2004, 2007, 2011, 2012). This divided attention likely accelerated memory decay for the disaster entities among the higher-proficiency English learners, compared to the Japanese learners with lower English proficiency. This argument is evidenced by the finding that as the L2 proficiency increased, the response time for detecting the changes in the disaster agents became longer (Refer to Appendix V). The observed increase in the response time can be interpreted as a sign of hesitation in the decision making, which gave rise to a decreased recall of the previously encoded information.

## General discussion

In this section, we review our research questions and hypotheses based on the results above. We asked in the first research question whether there are cross-linguistic differences in recognition memory of event roles between Japanese and English speakers. We hypothesised that Japanese speakers would better memorise human patients than English counterparts and English speakers would better memorise non-human agents than Japanese counterparts, especially for the animate non-human agents. The result was consistent with only the first half of the hypothesis. Japanese participants were indeed more accurate in remembering the human patients; accuracies in *PatientUnmatch* trials were significantly higher for the Japanese speakers than for the English speakers for *animal->human* and *disaster->human* items. However, the result was against the second half of the hypothesis. English participants did not show any advantage in memorising the non-human agents to Japanese participants; the accuracies in *AgentUnmatch* trials were comparable between the two groups for animal->human and disaster->human items. Moreover, surprisingly, English participants were less accurate than Japanese participants in remembering the human agents, which was supposed to be cross-linguistically similar because the agency and animacy effects are integrated into the human agents.

The results cannot be well explained if we attribute the cross-linguistic differences to an “agency-or-animacy” issue. In other words, the agency effect and the animacy effect are in a trade-off relationship. Nonetheless, this does not appear to be the case for the current study. Compared to English participants, Japanese participants showed an animacy advantage in the recognition memory of human patients. At the same time, they also performed as accurately as the native English speakers in remembering the non-human agents (animals and disasters). This suggests that there may coexist agency and animacy effects in the Japanese language. That is, Japanese speakers are guided by the animacy effect without overly downplaying the importance of agents. This is probably because the agent, as the initiator of the action chain, is important for the existence of the whole event (Cohn & Paczynski, 2013; Kemmerer, 2012; Talmy, 1988). Even if agency is not prioritised as in the Japanese language, its role in helping grasp the gist of the event is crucial for the event perceiver. This assumption is further evidenced by a counterintuitive finding that, as the waiting time went by, Japanese participants’ response accuracy for the animal mismatch gradually increased, which contrasts with the memory decay of the animal agents observed in English participants (Refer to Appendix II). This implies that the agency effect gradually increased for Japanese participants as the information storage time became longer. Importantly, this line of arguments can help us account for the unexpected finding that Japanese participants responded to the mismatch of the human agents in the category human->animal more accurately than English participants. The additive effect of animacy and agency gave rise to an absolute advantage in the recognition memory of the human agents for Japanese participants and consequently a slower memory decay for the human agents compared to English participants (Refer to Appendix II). Nonetheless, this additive effect did not generate a significant advantage in remembering the human agents in the category of human->object for Japanese participants, though the Japanese speakers were slightly more accurate in detecting the change of human entities than the English speakers. A possible explanation is that the category of human->object is different from other categories in terms of time duration. Perceivers of events keep updating the memory representation of events at event boundaries of a temporal frame (Ditman et al., 2008; Radvansky & Zacks, 2011; Speer & Zacks, 2005). The temporal boundary of human-throwing objects is arguably different from the boundaries of the other categories. It usually takes only several seconds for the events of throwing objects to end, whereas the events of chasing last longer with fuzzier temporal boundaries and more steady internal states. Therefore, when perceivers view the events of throwing objects, it is possible that they update the memory representation more quickly than when viewing the events of chasing. When they update the mental representation, they shift the attention from the human agents to the object patients, because the event state has changed from the process of throwing to the result that the object is lying on the ground. This is supported by the finding that both groups of participants were more accurate in remembering the objects than the human entities (See Appendix I for the intra-language comparisons of the response accuracy for *AgentUnmatch* and *PatientUnmatch* trials across the four categories), which is reminiscent of the finding in several prior studies that patients play a key role in change-of-state events (Hindy et al., 2012, 2015; Ünal & Papafragou, 2019). Therefore, a more rapid update of the mental representation of the human agents arguably weakened the advantage of memorising the human agents for the Japanese speakers, thus shrinking the cross-linguistic differences between the two groups. This line of reasoning is further supported by the finding that the cross-linguistic differences between Japanese and English in memorising the human agents of *human->object* items became smaller gradually with longer waiting times, which is opposite to that for *human->animal* (See Appendix II).

The results here demonstrated that the similarity between the linguistic expressions and the mental representations of event roles is not strict (Ünal et al., 2021b, 2024). In other words, how event roles are encoded in language do not always faithfully reflect how event roles are conceptually represented. For example, in the present study, both the agency effect and animacy effect influenced Japanese participants’ recognition memory of event roles whereas the animacy showed an absolute advantage over agency in Japanese speakers’ linguistic data (See Qu & Miwa, 2024). Moreover, both English and Japanese participants were significantly more accurate in responding to the changes in the object patients than in the human agents (Refer to Appendix I), whereas both groups of speakers showed a strong tendency to select the human agents as the subjects of sentences in the description data of Qu and Miwa (2024). Furthermore, although the English speakers did not show any tendency to select the disaster agents as the subjects of sentences in Qu and Miwa (2024), the English speakers in the current study were more accurate in judging the changes in the disaster entities than in the human entities (Refer to Appendix I). Future studies should probe into the reasons for the observed inconsistency between language and cognition in event roles.

The coexistence of agency and animacy effects suggests that agents are relatively prominent even for speakers of a language that does not prioritise the agency, such as the Japanese language. Having said that, this effect does not point to the agency preference (the agency advantage) as a cognitive universal claimed in the previous linguistic studies (e.g., Grimshaw, 1990; Jackendoff, 1990; Pinker, 1989; Wilson et al., 2022) as well as the psycholinguistic studies (e.g., Gerwien & Flecken, 2016; Flecken et al., 2019; Isasi-Isasmendi et al., 2023; Sauppe et al., 2023; Wilson et al., 2011). In the current study, Japanese participants were still slightly more accurate in responding to the changes in the human patients than in the non-human agents whereas English participants were significantly more accurate in detecting the changes in the non-human agents than in the human patients, especially when the non-human agents are animate entities (See Appendix I). This is especially true if we take into consideration how the English learning experiences changed the patterns of the recognition memory of Japanese participants.

Apart from the coexistence of agency and animacy effects, L2 learning is also an important factor for explaining the observed cross-linguistic differences in the recognition memory of event roles between Japanese and English. In our second research question, we hypothesised that L2 learning experiences attenuate Japanese participants’ human-oriented perspective in the recognition memory of event roles. The results are consistent with our hypothesis. As the L2 proficiency increased, Japanese participants became more accurate in detecting the changes in the animal agents and less accurate in detecting the changes in the human patients as demonstrated in Fig. 4 (See also Appendix III for the similar results for the daily English exposure). The enhanced agency effect and attenuated animacy effect indicate that the reconceptualisation of event roles happened in the Japanese learners with more L2 (English) learning experiences. This finding is consistent with several previous studies reporting the cognitive restructuring mediated by L2 proficiency and language contact (e.g., Athanasopoulos, 2006, 2007; Ji, 2017; Bylund et al., 2013; Wang & Wei, 2019). Our result has extended the research on L2 cognitive restructuring by adding another piece of evidence for cognitive restructuring when learning different conceptual representations of event roles in L2. Taking into consideration the cognitive restructuring observed in the Japanese learners, we can argue that compared to the findings in the present study, the cross-linguistic differences between Japanese and English speakers would have been larger in terms of the response accuracies for the animal agents and the human patients if all the Japanese speakers had limited English proficiency. Specifically speaking, with lower English proficiency, Japanese speakers would show a larger animacy effect and a smaller agency effect, which would result in a higher response accuracy for the human patients and a lower response accuracy for the animal agents, compared to the present study. This outstanding animacy advantage over agency in the mental representations of event roles directly questions the universality of the agent-first hypothesis, at least at the level of recognition memory in which language is arguably used as a strategy for information encoding. The finding here is against the view that the conceptual knowledge of event categories is universal across speakers of different languages as implied in some previous studies (e.g., Gleitman & Papafragou, 2005; Ünal & Papafragou, 2016; Ünal et al., 2021a). Instead, it supports an alternative view that cross-linguistic differences give rise to cognitive biases when viewing the world (e.g., Boroditsky, 2001, 2006; Levinson, 2003). Nonetheless, it is still possible that the low-level perception of event roles is universal across speakers of different languages, in which linguistic effects have not fully emerged yet. This can be a topic for future research.

Interestingly, the L2 findings in the present study, based on the non-linguistic data, appear to be inconsistent with the L2 findings based on the linguistic data of Qu and Miwa (2024), which reported that Japanese learners of English had difficulty reconceptualising event roles in the L2 descriptions. The reason for the difference between the two studies cannot be attributed to L2 proficiency, for a t-test did not show any differences between participants of the present study and participants in Qu and Miwa (2024) in the self-reported English proficiency scores. More importantly, such disparity contrasts with the findings of many previous studies on the relationship between L2 linguistic data and L2 cognitive data, which demonstrated an opposite pattern that the cognitive restructuring is more difficult to achieve than linguistic restructuring due to the dissociated learning of L2 linguistic structures from their cognitive functions (Jarvis & Pavlenko, 2008; Pavlenko, 2014). A possible interpretation is that although the Japanese learners of English had difficulty acquiring the English event roles at the linguistic level due to the low noticeability of the probabilistic differences in linguistic encodings of the relational information (agents and patients), the Japanese learners of English arguably received more input on the perspective of event construal from various linguistic features in L2 English (not limited to the event roles), which might have driven the Japanese speakers to take a more agent-focused perspective (objective construal) in conceptualising the event roles at the cognitive level. For example, Qu and Miwa (2024) argued that a large number of transitive verbs in English and the absence of the semantic distinction between “ego” and “others” in English give-type benefactive constructions all motivate Japanese learners of English to acquire an external perspective in viewing events. Therefore, this study calls our attention in language and cognition research to how a single linguistic structure might relate to other structures in a language (Enfield, 2015).

In general, our findings have broader cognitive implications for the psychological science. The stronger animacy effect in the Japanese language can potentially influence sentence comprehension of Japanese speakers. Past studies on sentence processing have suggested that agents come first during incremental processing. That is, readers tend to interpret the first noun that they encounter in a sentence as an agent argument (Bader & Meng, 1999; Dröge et al., 2020; Haupt et al., 2008; Krebs et al., 2018; Meng & Bader, 2021), even in a patient-first language such as Äiwoo (Sauppe et al., 2023). However, the case of Japanese might provide counterevidence for this long-standing argument. Because of the stronger animacy effect over the agency effect, subjects of sentences are less frequently assigned agent arguments, especially in the cases where the most animate entities are patients, such as in the images showing animals chasing humans (Qu & Miwa, 2024). This attenuated agency preference even extends to human-only events. For example, Ito (2016) reported that Japanese speakers were more likely to select human patients as subjects than English speakers in describing the events showing human agents acting upon human patients, such as a police officer is arresting a man. Therefore, Japanese speakers, influenced by usage frequency, may show a weakened tendency to interpret the first noun of a sentence as an agent. The Japanese case is of importance to test the universality of the agent-first preference in sentence comprehension.

Moreover, as the strong animacy effect is rooted in the preference for a subjective perspective, our results also have close connections with the cognitive psychology studies on visual narratives. For example, Cohn (2011), Cohn et al. (2012), and McCloud (1993) found that Japanese manga use a subjective view in the narratives whereas American comic books use an objective view. That is, English manga tend to use more full scenes to present a third-person perspective, whereas Japanese manga tend to use more close-up scenes to focus on the actions of the main characters in the narratives, with the purpose of inviting the reader to have an immersive mental experience during the manga reading. This suggests that Japanese speakers are prone to project themselves onto the scenes to be construed by taking an internal perspective, but English speakers are prone to detach themselves from the scenes to be construed by taking an external perspective. Furthermore, our results are highly associated with the psychological studies on accuracy and vividness in autobiographical memory. For example, some studies showed that autobiographical memory retrieved from an observer perspective (the external vantage point) can be less accurate and vivid than retrieved from an immersed first-person perspective (e.g., Brédart & Bouffier, 2016; Dranseika et al., 2021; Marcotti & St. Jacques, 2018), which seems to echo with the finding of the present study that compared to English participants, Japanese participants had more accurate memory of the human entities and showed no disadvantage in memorising the non-human agents. Future research can explore whether there are cross-linguistic differences in autobiographical memory between Japanese and English or other languages that prefer an objective perspective.

Finally, we present several limitations of this study for future research. First, testing participants online might have introduced more noise in the data due to the differences in individual devices and environments for conducting the experiment. English participants recruited through Amazon Mechanical Turk might have lacked the motivation to take the task seriously because of the anonymity issue (Peer et al., 2022), though this is one of the common practices in collecting participants as done in many past studies (e.g., Enochson & Culbertson, 2015; Schmidtke & Kuperman, 2024). Future studies should take these methodological issues into account. Second, our task was relatively easy to handle for participants (the average accuracy for *AgentUnmatch* trials was 87.5% and 88.4% for *PatientUnmatch* trials). A lower accuracy can provide us with more variance in data for the error analysis to better detect the effect of independent variables. Future studies can increase the difficulty of the task to press the memory of Japanese and English speakers to see whether the cross-linguistic differences found in this study are replicable. Third, this study adopted the TFS hypothesis (Slobin, 1996) as the theoretical framework to explore the interface of language and cognition. To this end, we designed the experiment in the way that language was potentially used as a strategy for encoding and storing information (e.g., the randomised image presentation time and the waiting time). Therefore, our results can only be interpreted as the language-specific effects on recognition memory, as argued in some previous studies (e.g., Gennari et al., 2002; Koster & Cadierno, 2019). Whether language exerts its effect on cognition at a deeper level in the domain of event roles remains unknown. Lastly, there may be doubts over whether cultural factors also played a role in the observed cross-linguistic differences between Japanese and English. Indeed, the strong animacy effect in Japanese likely reflects a culturally grounded empathy bias. This might originate from the importance of interpersonal relationships in East Asian cultures, where relationships are often hierarchically structured. In a culture that has high interpersonal sensitivity, projecting oneself onto others to better understand others’ feelings is necessary. Future research can test whether culture or language plays a bigger role by comparing participants from other East Asian countries, such as Korea and China, with Japanese participants.

## Conclusion

Using event roles as a window onto the perspective of event construal, this study explored whether the cross-linguistic differences in the preferred perspective of event construal influence the recognition memory of event roles. This is the first study that reported positive evidence for linguistic effects on recognition memory of event roles, which challenged the universality of the conceptual knowledge of event roles among speakers of different languages. Guided by the subjective construal, the Japanese speakers tended to prioritise animacy over agency, which motivated them to memorise the human patients more accurately than the English speakers did. At the same time, the Japanese speakers considered agency as well, which gave rise to the coexistence of agency and animacy effects. This additive effect motivated the Japanese speakers to memorise even the human agents more accurately and showed no disadvantage in memorising the non-human agents compared to the English speakers. Furthermore, this study found that cognitive restructuring happened in Japanese learners of English, which was mediated by their L2 proficiency.

## Figures and Tables

**Fig. 5:**
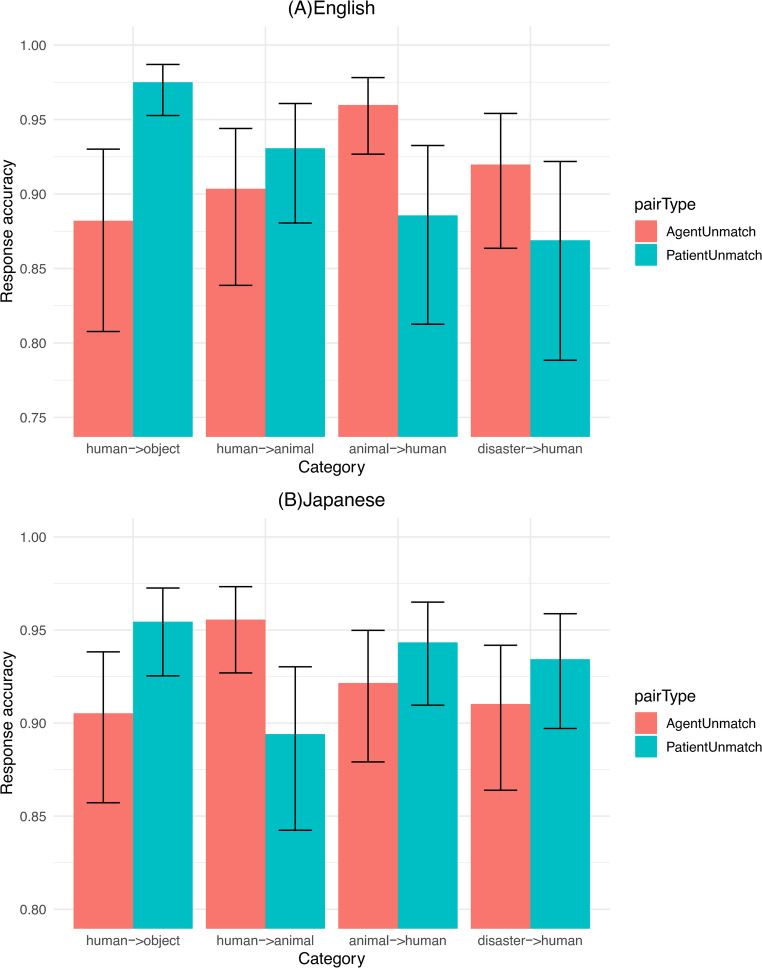
Response accuracies of *AgentUnmatch* and *PatientUnmatch *by English (Panel **A**) and Japanese (Panel **B**) language groups across the four categories of images

**Fig. 6:**
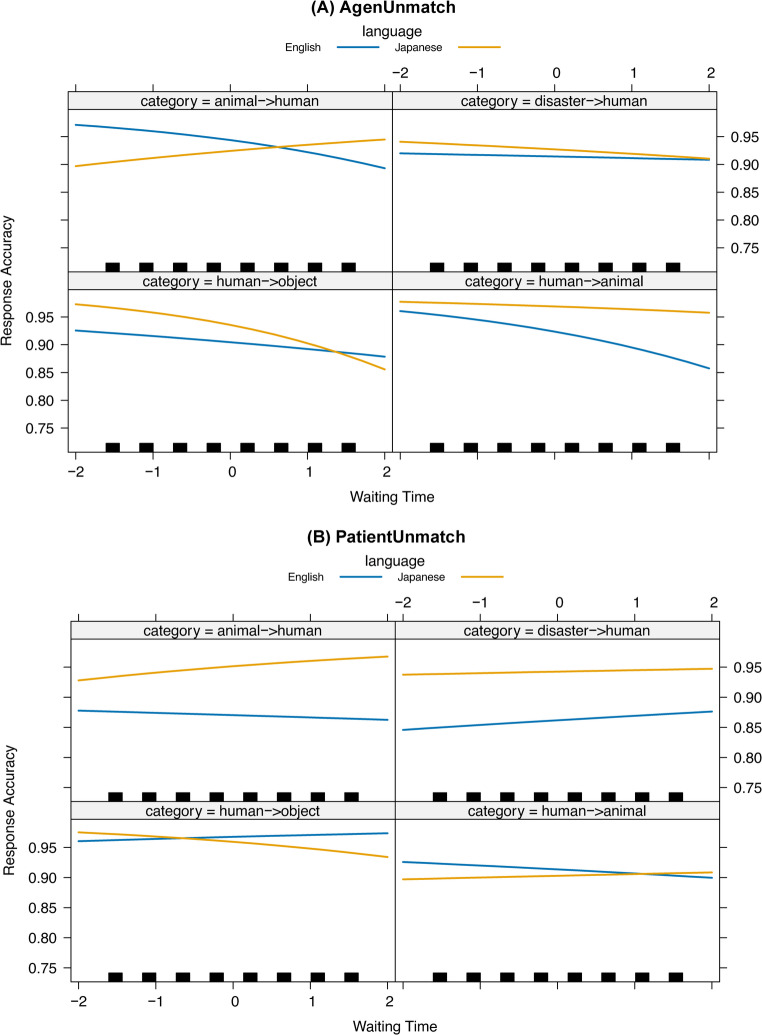
The interaction for response accuracies of *AgentUnmatch* (Panel **A**) and *PatientUnmatch* (Panel **B**) between waiting time, language, and category

**Fig. 7:**
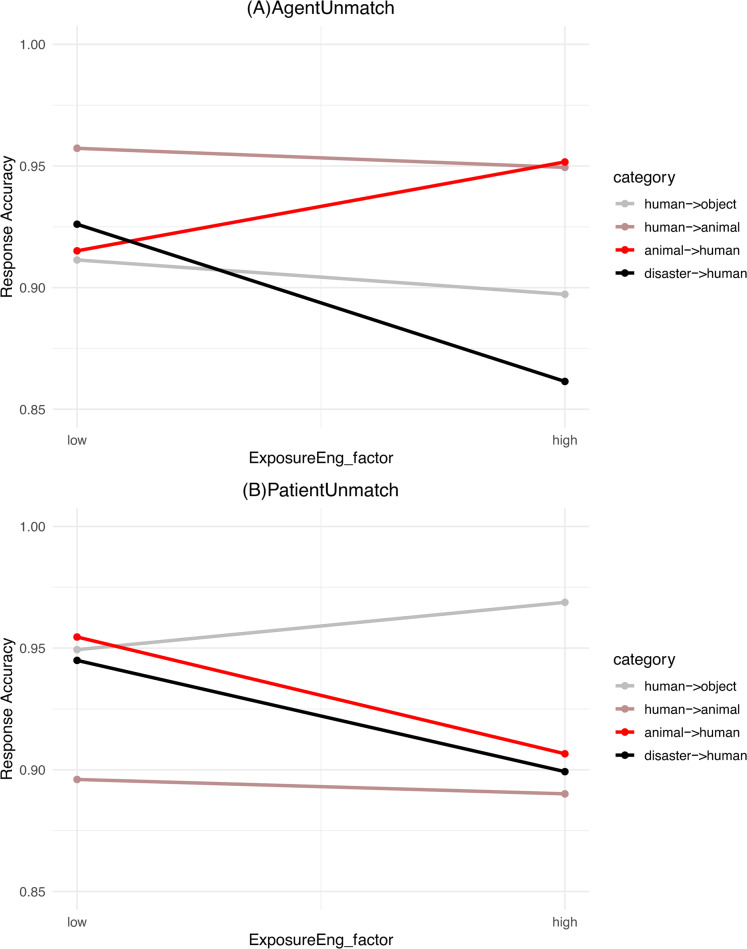
Japanese participants’ response accuracies mediated by English exposure in *AgentUnmatch* (Panel **A**) and *PatientUnmatch* (Panal **B**) across the four categories of images

**Fig. 8:**
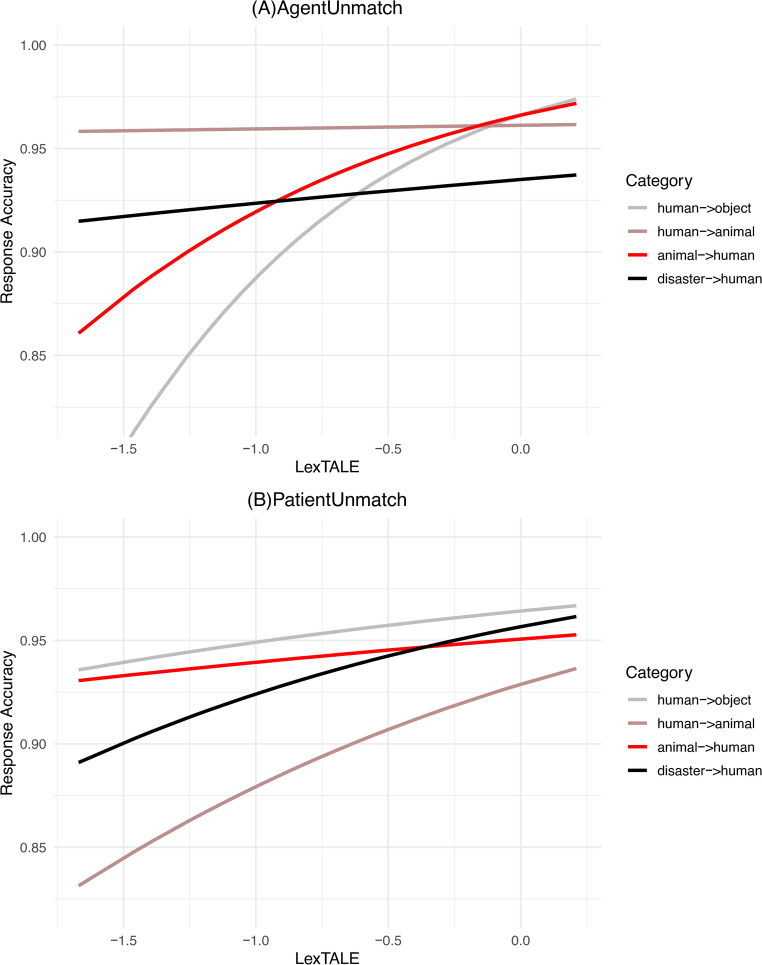
Japanese participants’ response accuracies mediated by LexTALE scores in *AgentUnmatch* (Panel **A**) and *PatientUnmatch* (Panel **B**) across the four categories of images

**Fig. 9:**
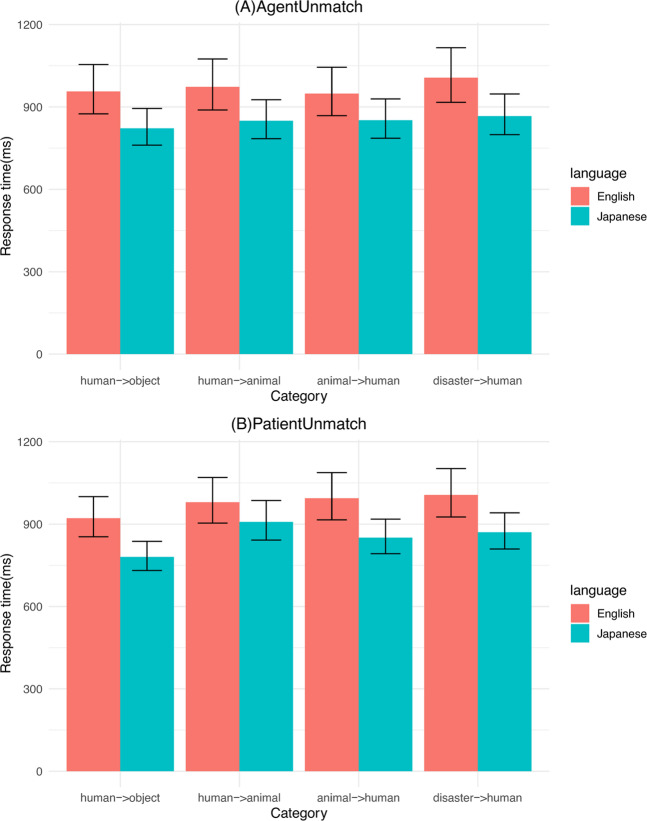
Reaction times for *AgentUnmatch* (Panel **A**) and *PatientUnmatch* (Panel **B**) by the two language groups across the four categories of images

**Fig. 10:**
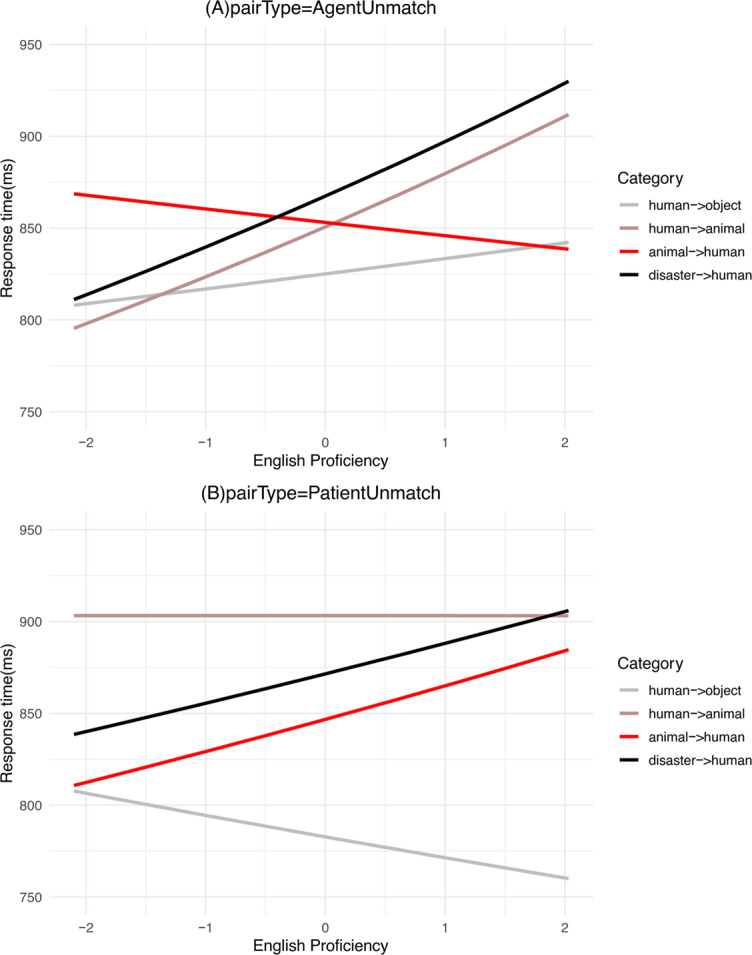
Japanese reaction time mediated by English proficiency in *AgentUnmatch* (Panel **A**) and *PatientUnmatch* (Panel **B**) across the four categories of images

**Fig. 11:**
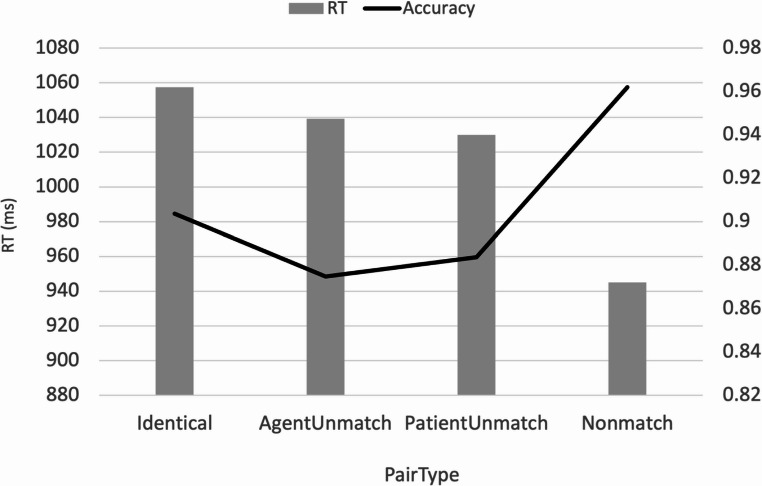
Comparisons of reaction time and accuracy across the four *pairType*

**Fig. 12:**
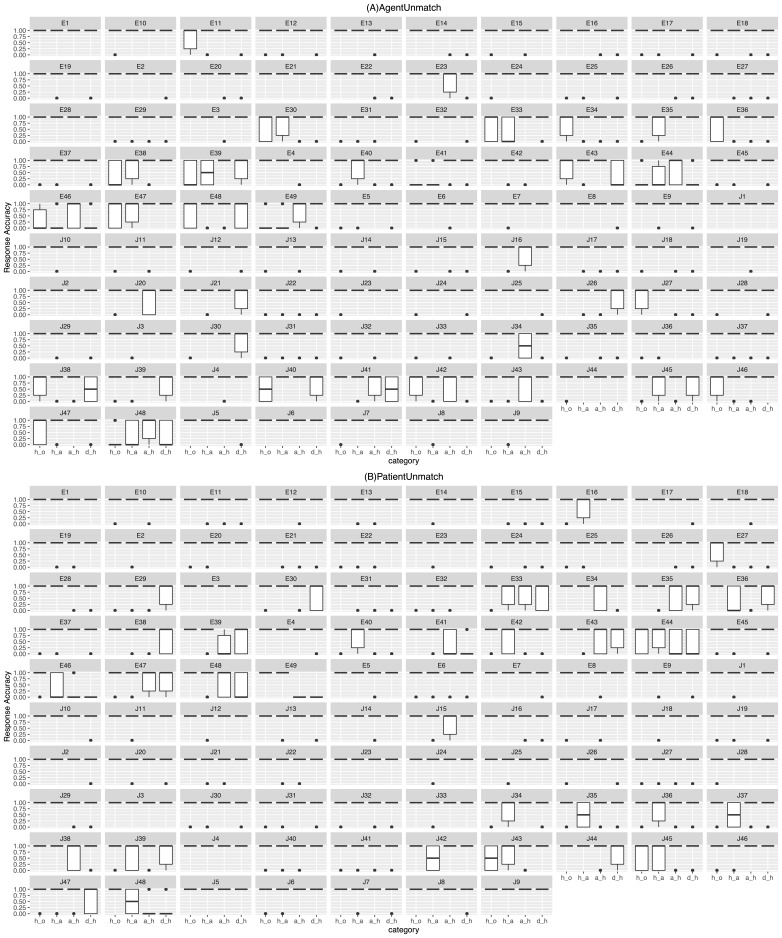
Variance of participants’ response accuracies in *AgentUnmatch* (Panel **A**) and *PatientUnmatch* (Panel **B**) across the four categories of images (the category names are shortened). “J” stands for Japanese and “E” English

## Data Availability

The original data, the R codes and an R Markdown file used for the data analysis are all publicly available on the Open Science Framework: [https://osf.io/q2eyf/files/osfstorage](https:/osf.io/q2eyf/files/osfstorage).

## Appendix III

The predictor *exposureEng_factor* is a categorical variable that measures the participants’ length of contact with English on daily basis. This predictor was created based on the bigram distribution of the average scores of six self-reported ratings on daily English use in the LEAP-Q: daily English use with friends, family, TV, radio, reading books, and language classes. If the average score of daily English use was larger than 3, the exposure to English would be labelled “long,” vice versa for the short cases. We chose to dichotomise this predictor because more than half of Japanese participants rated “0” on daily English use. The predictor *exposureEng_factor* showed a significant interaction with the category in *AgentUnmatch* condition and a marginally significant interaction with the category in *PatientUnmatch* condition (See Fig. 7). Frequent exposure to English has weakened the human-oriented perspective of Japanese speakers but strengthened the agent-focused perspective of Japanese speakers in animal->human. The results here pattern with the self-reported average English scores (see Fig. 4) to a large extent, suggesting that frequency of language use is closely related with general proficiency in that language (Bylund et al., 2010). The results of exposure to English further indicate that language contact is an important indicator for predicting cognitive restructuring in L2 (Athanasopoulos et al., 2011; Bylund et al., 2013; Wang & Wei, 2019). Frequent usage of one language can strengthen the language-specific associations and thus change mental representations (Bylund & Athanasopoulos, 2014).

Moreover, *LexTALE_score* showed a significant interaction with *category* in *AgentUnmatch* and a non-significant interaction in *PatientUnmatch* (See Fig. 8). The results demonstrated that the Japanese learners with higher LexTALE scores tended to be more accurate in responding to the change in both agents and patients. This finding, not consistent with any of our hypotheses, seems to be predicting the verbal working memory of participants and the seriousness of participation in the experiment rather than L2 proficiency. Compared to the results based on the self-rated English proficiency and the daily exposure to English, the finding with the LexTALE scores suggests that LexTALE may have not accurately measured the L2 learners’ proficiency, for it may be more useful as a predictor of vocabulary knowledge than that of general proficiency, especially for non-advanced learners of English (Nakata et al., 2020). This is evidenced by a weak positive correlation with the average self-reported English scores (*r* =.251, *p* =.085).

The predictor *foreign_experience* is a categorical variable that measures whether participants had the experience of living in English-speaking countries (*yes*) or not (*no*). This predictor did not show any significant interactions with Japanese participants’ response accuracy. A possible reason is that only a small number of Japanese participants had the experience of living in English-speaking countries (13 out of 48).

## Appendix IV

Except for the category of human->animal in Panel (B), Japanese participants responded significantly faster than English participants in all the rest of the categories for both *AgentUnmatch* and *PatientUnmatch*. There are at least two possibilities that can help explain the better performance of Japanese participants. First, Japanese participants’ average digit span scores were higher than those of English participants (Japanese mean: 6.73, English mean: 5.47, *p* <.001). This suggests that the Japanese group, in general, had larger verbal working memory than the English group, though the predictor *digit_score* was not significant in the models for RT. The larger verbal working memory might have facilitated recollection of the event roles for Japanese participants. Second, the Japanese and English speakers participated in the experiment in different ways: Japanese participants disclosed their personal information whereas English participants remained anonymised. As mentioned in the limitation part, the anonymity issue might have demotivated English participants to take the task seriously (Peer et al., 2022), thus giving rise to the longer RTs.

The small cross-linguistic difference between the two groups in the category of human->animal in *PatientUnmatch* was caused by the relatively slower response time of Japanese participants compared to the RTs of Japanese participants in the rest of the three categories. This result is reminiscent of the finding in the accuracy analysis, which showed that in human->animal, Japanese participants were significantly more accurate than English participants in detecting the change in the human entities (See Fig. 3). We argued in the discussion part that there may coexist agency and animacy effects for Japanese speakers, so the human entities in human->animal had an absolute advantage in the mental representations. This explains why Japanese participants had difficulty in retrieving the memory of the animal patients, which resulted in longer RTs.

## Appendix VI

The figure above shows that *Identical* took the longest time for participants to respond to and *Nonmatch* took the shortest time, which is suggestive of the participants’ response strategy in the experiment. That is, participants looked for the differences between the two images rather than directly mapping the memory of Image 1 with Image 2. If they found any differences, they pressed the “no” button. Otherwise, they pressed the “yes” button.

## Footnotes

1
*Nonmatch* can provide us with a baseline for comparison with *AgentUnmatch* and *PatientUnmatch*. If participants processed both the agents and the patients in the stimuli, the response accuracy for *Nonmatch* would be higher and the response times would be faster than *AgentUnmatch* and *PatientUnmatch* (See Appendix VI).

2 The interactions between *language* and *category* were non-significant for *Identical* (9764 data points) and *Nonmatch* (1949 data points), suggesting that the cross-linguistic differences found in *AgentUnmatch* and *PatientUnmatch* were condition-specific effects.

3 We standardised the continuous variables to facilitate the interpretation of regression coefficients and to ensure comparability across predictors on different scales. Standardisation was performed using z-scores (mean = 0, *SD* = 1) prior to model fitting.

## Declarations

### Public Significance Statement

This study demonstrates that the preferred perspective of a language influences how its speakers remember event roles (agents and patients), challenging the assumption that conceptual representations of event roles are universal across languages. This study offers new insights into the relationship of language and thought and has implications for legal settings of eyewitness testimony.
